# Supplementary material for: The interactome of intact mitochondria by cross-linking mass spectrometry provides evidence for coexisting respiratory supercomplexes
Source: Mol Cell Proteomics. 2017 Dec 8;17(2):216–32. doi: 10.1074/mcp.RA117.000470 (PMC5795388; doi:10.1074/mcp.RA117.000470)
Supplement: Supplemental Data [file supp_RA117.000470_133922_0_supp_23978_fzffwf.zip › spectra_annotation/mito_DR_spectra_annotation/36-1-3-1-9-1.pdf]

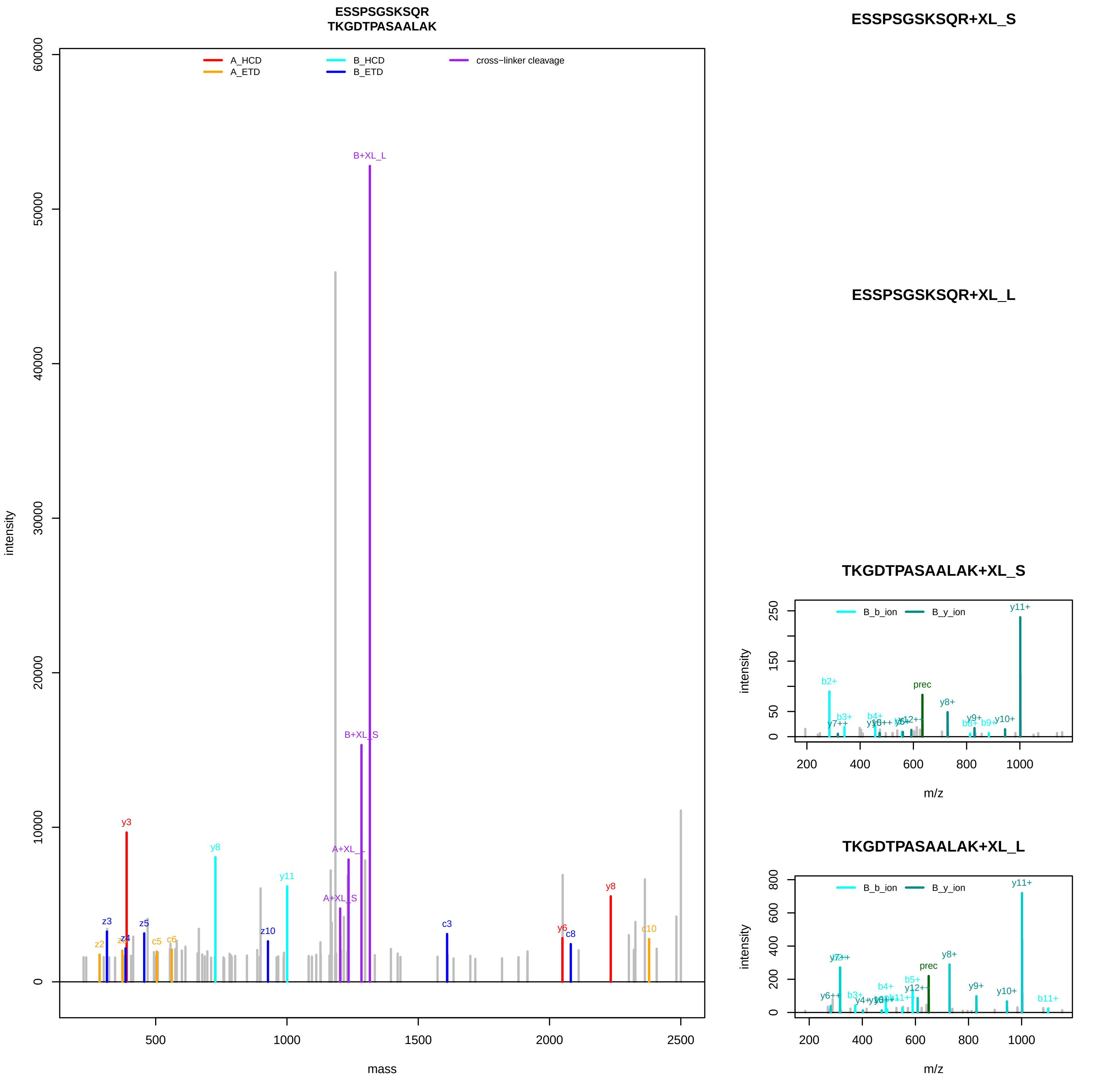

ESSPSGSKSQR
TKGDTPASAALAK
A_HCD
A_ETD
B_HCD
B_ETD
cross−linker cleavage
intensity
mass
B+XL_L
B+XL_S
A+XL_L
A+XL_S
y3
y8
y11
z2
z3
z4
z5
z10
c5
c6
c3
c8
c10
y6
0
10000
20000
30000
40000
50000
60000
500
1000
1500
2000
2500
ESSPSGSKSQR+XL_S
ESSPSGSKSQR+XL_L
TKGDTPASAALAK+XL_S
TKGDTPASAALAK+XL_L
B_b_ion
B_y_ion
prec
y11+
y8+
y9+
y10+
b2+
b3+
b4+
b5+
b11+
y12++
y7++
y6++
m/z
200
400
600
800
1000
